# The Role of New Agents and Supportive Care in a Multimodal Approach to Cancer Cachexia

**DOI:** 10.3390/cancers18040649

**Published:** 2026-02-17

**Authors:** Egidio Del Fabbro, Sudeep Pandey

**Affiliations:** 1 Division of Palliative Medicine, Department of Medicine, Medical College of Georgia, Augusta University, Augusta, GA 30912, USA; 2 Division of Hematology, Oncology and Palliative Care, Virginia Commonwealth University, Richmond, VA 23284, USA; sudeep.pandey@vcuhealth.org

**Keywords:** cachexia, multimodal, symptoms, GDF-15, anabolism, clinical trials

## Abstract

Given the multi-faceted nature of cancer cachexia, a combination of pharmacologic and supportive measures such as exercise and nutrition seems intuitive to most clinicians. Clinical trials have also suggested that a multimodal approach to cancer cachexia (CC) is feasible and potentially effective. However, past trials have been limited by medications that were partially effective or had the potential for serious adverse events such as thromboembolism. We review new agents that have demonstrated efficacy in phase II trials or are involved in multi-center phase III studies. The advent of several recent phase II studies indicate that consistently effective, well-tolerated medications may soon be available for CC. These new agents target several mechanisms involved in CC, including food aversion, catabolism, and decreased anabolism. Several multi-center studies are expected to be actively recruiting this year. If these agents prove to be effective, individualized treatment may be possible, guided by individuals’ phenotype and/or clinical biomarkers. Future research should also determine whether combination therapy (pharmacologic and/or non-pharmacologic) produces additive or synergistic benefits.

## Simple Summary

There is an unmet need for effective treatments in patients with cancer cachexia. Patients suffering from cachexia endure weight loss, fatigue, poor appetite, a diminished quality of life and decreased survival. There are no medications approved for this condition in North America or Europe. Fortunately, several recent studies show that effective treatments improve appetite and are well tolerated with few side effects. These treatments will be evaluated in large multi-center trials within the next year. In future, these medications may prove to be even more effective when combined, or added to supportive care measures such as nutrition, exercise, and psychological counseling.

## 1. Introduction

Cancer cachexia is associated with decreased survival, poor tolerance to anti-neoplastic therapy and diminished quality of life. The overall prevalence of cachexia is estimated to be 33.0% but varies depending on the criteria used and the definition of cancer cachexia [1]. Yearly estimates for patients suffering from cancer cachexia total 527,100 in the USA, and 800,300 in the European Union [2]. The magnitude of cachexia’s impact and its contribution to adverse clinical outcomes underscores the need for effective therapies. Based on recognition of the multifactorial contributors to the cachexia–anorexia syndrome and their mechanistic underpinnings, there is sustained interest in combining pharmacological and non-pharmacological therapies for cancer cachexia. Clinical trials have also highlighted the potential value of multimodal therapy. In 2010, Mantovani et al. showed, for the first time, that a multimodal regimen is more effective for cachexia-related outcomes than any of its individual components [3]. The combination of medications and nutritional supplements improved several cardinal features of cachexia including lean body mass, spontaneous physical activity and appetite [3]. The most recent international multimodal trial (MENAC) combined nutrition, exercise, anti-inflammatory drugs and anti-neoplastic therapy, resulting in modest weight stabilization but no significant improvements in muscle mass or activity [4]. Given the advent of new agents showing remarkable efficacy in early phase trials, it is reasonable to consider how these new therapies might be effectively combined with interdisciplinary supportive care, optimal symptom management and hormone replacement therapy. Multimodal therapy could produce additive or synergistic benefits.

The aim of this narrative review is to identify new agents with demonstrated efficacy in phase II trials, or that are being evaluated in active, multi-center phase III studies. Their potential for improving clinical outcomes as individual agents or in multimodal therapy are discussed in further detail. Older cancer cachexia studies yielding promising results included medications such as betablockers or insulin [5] in combination with nonsteroidal anti-inflammatories. Although important clinical outcomes such as weight and survival improved, the methodology was limited by its use of no placebo control. In addition, the contributions by symptom management and interdisciplinary support were either omitted or not evaluated in trials using pharmacologic agents. An exception is an open-label phase III trial published in 202 demonstrating the value of non-pharmacologic therapy coupled with anti-neoplastic treatment [6]. Early interdisciplinary care focused on *dietary* and *psychological* aspects and resulted in weight gain, increased tolerability of chemotherapy cycles and improved survival compared to standard care [6].

**Figure 1:**
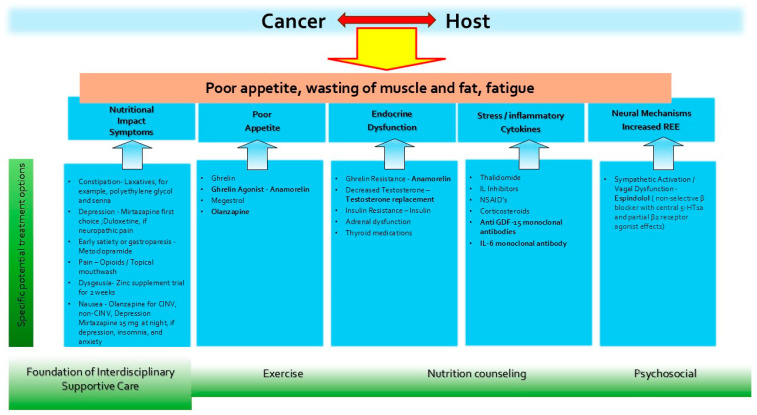
Theoretical model for multimodal cancer cachexia therapy. GDF-15 (growth/differentiation factor 15); IL-6 (interleukin 6); and CINV (chemotherapy-induced nausea and vomiting).

Figure 1 shows a framework for the multimodal management of cancer cachexia, including the various agents under investigation. Interventions with evidence for efficacy in phase II or phase III trials are bolded in Figure 1. Although our review focuses on pharmacologic interventions, interdisciplinary care encompassing exercise, nutrition and psychosocial support is integral to any multimodal approach.

## 2. Methods

### Search Strategy

The purpose of this narrative review is to identify agents for cancer cachexia that demonstrated efficacy in phase II studies or are being tested in actively recruiting phase III multi-center studies. We restricted our search to studies published in PubMed or listed in Clinical Trials.gov from 2016 until 2026. A literature search using PubMed with the following keywords ‘phase III cancer cachexia trial’ or ‘phase II cancer cachexia trial’ yielded 48 and 56 results, respectively.

The search also included Clinical Trials.gov with terms cachexia (510 studies), ‘cancer cachexia’ (259), and ‘sarcopenia in cancer ‘ (118), while cancer and fatigue identified 90 studies when ‘drug treatment’ was added to the search term.

Only phase II or phase III intervention studies were evaluated by both authors. Their inclusion in the final review was based on potential efficacy indicated by phase II studies achieving improved cachexia related clinical outcomes. The nine studies identified are summarized in Table 1. Excluded from the review were phase I studies or published phase II/III studies without an improved clinical outcome. Studies recruiting participants without cancer were also excluded.

## 3. Inflammatory and Somatic Stress Cytokines

Pre-clinical research shows that pro-inflammatory cytokines such as tumor necrosis factor α (TNF-α), IL-6 and interferon gamma play a prominent role in the pathogenesis of CC. Other cytokines such as myostatin and growth/differentiation factor 15 (GDF15) were subsequently identified as having targetable roles in CC and evaluated in clinical trials. These cytokines produce a cascade of interrelated metabolic changes, including proteolysis, lipolysis, lipolysis, lipid mobilization, increased resting energy expenditure, decreased muscle synthesis, lipogenesis, and anorexia. Trials with agents targeting individual cytokines such as TNF-alpha [7] or IL-beta [8] were disappointing or partially effective and not pursued in larger trials. However, IL-6 and, especially, GDF-15, exhibit potential as biomarkers of cachexia and as therapeutic targets. Recently, a randomized phase II trial in patients with advanced pancreatic cancer compared chemotherapy treatments with and without an IL-6 receptor antibody (tocilizumab). Improved overall survival at 18 months and reduced muscle wasting were identified in the group receiving IL-6 blockade [9]. The GDF-15 levels were unchanged, suggesting an anti-cachectic effect, independent of GDF-15.

### Anti-GDF-15 Agents

In healthy individuals, GDF-15 expression is predominantly in the placenta [10], followed by the prostate. The normal range in healthy adults is broad (200–1200 pg/mL), with higher levels strongly associated with chronological age [11]. GDF-15 also exhibits a diurnal variation, and is upregulated by mitochondrial dysfunction and secreted by a variety of cells in response to stressors [12], such as myocardial ischemia and by drugs such as metformin [13] and cisplatin. GDF-15 plasma levels are higher in patients with cancer-related weight loss and are associated with weight loss, sarcopenia [14] and worse survival in colorectal cancer [15]. Since anorexia is induced via the brainstem-restricted GDF-15 receptor GFRAL (glial cell-derived neurotrophic factor [GDNF] family receptor α-like), the blockade of this pathway is an appealing therapeutic target. A phase II, randomized, double-blind, 12-week trial administered a GDF-15 monoclonal antibody (ponsegromab) at doses of 100 mg, 200 mg, 400 mg or placebo subcutaneously every 4 weeks [16]. Improvements in weight, appetite, quality of life, body composition and physical activity were reported in the 400 mg ponsegromab group relative to placebo. Remarkably, patients with very poor metabolic reserve who may have been considered refractory to anti-cachexia therapy in the past had a significant response at 12 weeks. A large phase II/III multi-center RCT is underway in patients with pancreatic cancer and CC, including outcome measures of body weight, anorexia scores, overall survival, spontaneous physical activity and body composition.

Anti-GDF-15 agents are likely pleotropic and may have effects on multiple cell types since GDF-15 can be induced in most cell types in response to stress, including cancer cells. An intriguing, anti-neoplastic effect appears to be mediated by visugromab, another agent in the class [17]. An early phase trial in patients with advanced cancer, refractory to checkpoint inhibitors (anti-PD-1 or anti-PD-L1) found the addition of visugromab produced remissions in about one in six patients. More than half the responders achieved a confirmed complete metabolic response (as per PET-CT). Given that neutralizing GDF-15 may overcome resistance to immune checkpoint inhibition in cancer, the improvements in cachexia-related outcomes may be mediated by more than one pathway, e.g., including an anti-neoplastic effect. A multi-center, international, phase II/III trial of visugromab in patients with solid tumors and cachexia is planned for 2026, with weight and appetite as the primary outcomes. Neither ponsegromab nor visogromab mandate high levels of GDF-15 for eligibility in their phase II/III trials, despite the earlier phase II ponsegromab study requiring elevated GDF-15 (1500 pg/mL).

## 4. Anabolic and Anti-Catabolic Agents

### 4.1. Testosterone Replacement in Men (And Possibly Women)

Cachexia, characterized by weight loss, altered body composition, and decreased muscle mass/performance is particularly common in men with gastrointestinal malignancies. Males with cachexia have greater weight loss or muscle wasting [18] compared to females [19], and worse clinical outcomes for strength, fatigue and survival. This sexual dimorphism [20] may be explained by variances in muscle fiber types, muscle mitochondrial composition, and muscle gene expression patterns. In addition to cachexia-specific adverse outcomes, patients with low testosterone suffer from symptoms traditionally considered unrelated to cachexia, such as sexual dysfunction and depressed mood [21]. While these symptoms associated with hypogonadism may impair patients’ quality of life, they may also contribute to decreased nutritional intake. The causes of low T in patients with cancer include chronic inflammation, malnutrition, aging, radiotherapy, and medications such as opioids. More than 50% of males on opioids for chronic pain have low T, and the morphine equivalent daily dose is inversely correlated with serum testosterone levels in men with advanced cancer [22]. Several anti-neoplastic agents and glucocorticoids decrease testosterone. Alkylating agents decrease testosterone in a dose-dependent manner, while the lowering of T occurs especially rapidly with the tyrosine kinase inhibitor, crizotinib [23].

Inflammation, long thought to be a primary driver of cancer cachexia, is also associated with hypogonadism, and free testosterone levels correlate inversely with IL-6 levels in childhood cancer survivors [24]. GDF-15 has unclear effects on testosterone in oncology patients. However, in non-cancer patients with major depression, elevated GDF-15 levels are inversely associated with testosterone levels and correlate with the severity of depression in patients [25].

### 4.2. Clinical Outcomes

TRT improves body composition in young cancer survivors (aged 25–50) [26], with decreased truncal fat and increased lean body mass in men receiving TRT compared to placebo. There is also preliminary evidence for *supplemental* intramuscular testosterone improving lean body mass and physical performance in patients with advanced cancer. A double-blind, placebo-controlled trial in ten men and eleven women, with head and neck or cervical cancer compared 100 mg testosterone enanthate to placebo for 7 weeks. LBM increased by >3% in the testosterone arm, while both groups had similar declines in fat mass. The testosterone group experienced improved physical performance with a clinically meaningful increase in the SPPB score [27]. However, no significant changes were identified in terms of quality of life, and scores relating to muscle strength (leg extension), REE or survival.

Testosterone may improve other symptoms relevant to overall quality of life. Improved libido and erectile function [28] are potential advantages compared to other anabolic agents including selective androgen modulators. TRT also increases insulin sensitivity and may increase red cell production. However, patient expectations regarding the rapidity of TRT effect may need to be tempered. Fatigue scores were improved by day 72 of fatigue but not day 28 in a trial of male patients with advanced cancer, comparing TRT to placebo injections [29]. Outcome measures improving earlier (day 28) included libido and performance status.

### 4.3. Adverse Effects

Since TRT is contra-indicated in metastatic prostate cancer, there may be concerns that TRT also promotes non-hormone responsive cancers or precipitates thromboembolism, stroke or cardiovascular disease. On the contrary, some studies demonstrate inverse associations between testosterone levels and the risk of cancer. For example, men with higher levels of DHEA estradiol and testosterone are associated with a decreased risk of developing adenocarcinoma of the esophagus [30]. A large multi-center, double-blind, placebo-controlled, noninferiority trial that enrolled 5246 symptomatic, hypogonadal men 45 to 80 years of age with preexisting or a high risk of cardiovascular disease found TRT was noninferior to the placebo with respect to major cardiac events including death, non-fatal myocardial infarction and non-fatal stroke [31].

### 4.4. Indications and Formulations for Replacement

Although there is limited evidence for enhancing appetite or weight, TRT could target some important aspects of cancer cachexia such as sarcopenia or fatigue. Identifying appropriate patients with a convenient biomarker (low free or total T levels) would increase the likelihood of improved outcomes and decrease the risks of adverse events, given replacement levels would be limited to physiological levels. Similarly to other conditions characterized by chronic inflammation, oncology patients often have markedly elevated SHBG levels, which increases TT levels [32]. Free testosterone or bioavailable testosterone concentrations are a more reliable indicator of hypogonadism in cancer. The formulations of T include a transdermal patch, an alcohol-based transdermal gel, intramuscular injection and intranasal spray. Clinicians should adhere to Endocrine Society guidelines regarding target levels (mid-normal range for young men) for TRT and the monitoring of potential side effects The physiological levels for women are less certain.

### 4.5. Anamorelin

Although anamorelin is effective for several cachexia-related outcomes, it is unfortunately only approved in Japan, and its future global use is likely to be limited. In addition to the large ROMANA trials showing benefits in terms of improved weight, muscle mass, and body composition [33] in patients with cachexia, additional trials since then have supported its excellent safety profile, with mild hyperglycemia being the only consistently reported adverse effect [34]. Following approval in Japan, a multi-center, open-label randomized controlled trial in patients with unresectable or recurrent gastric cancer receiving chemotherapy were randomized to oral anamorelin 100 mg daily for 12 weeks or no anamorelin [34]. Although no significant difference was observed between the two groups, anamorelin showed a trend toward increased LBM with good tolerability and safety. A systematic review and meta-analysis of RCT’s including 1331 participants concluded that anamorelin produced a significant increase in body weight, lean body mass, fat mass in patients with cancer cachexia [35].

### 4.6. Espindolol

The activation of the sympathetic nervous system is implicated in the pathogenesis of weight loss for several conditions associated with wasting. A beta-adrenergic blockade with carvedilol attenuates the development of cachexia and promotes the partial reversal of cachexia in patients with heart failure [36]. In children with burns, propranolol attenuates hypermetabolism and muscle–protein catabolism [37], while in adults with burns, propranolol accelerates wound healing [38].

Twenty-five years ago, the use of betablockers for reducing resting energy expenditure was shown to be effective in a small group of patients with solid tumors and progressive weight loss [39]. Despite the promise of efficacy, there were no placebo-controlled trials with betablockers until a phase II trial compared to doses of espindolol to a placebo. In addition to the anti-catabolic effect of non-selective beta blockade, espindolol is thought to exert pro-anabolic, and appetite-stimulating effects through partial β2 agonism and central 5-HT1α receptors. The multi-center study in patients with colorectal cancer or NSCLC (stage III or IV) found that high-dose espindolol (10 mg twice daily) significantly increased lean body mass and hand grip strength. Plans for an additional phase II study followed by an international phase III trial are underway.

## 5. Nutrition Impact Symptoms and Interdisciplinary Supportive Care

Although many of the promising new agents for cachexia appear to have broad benefits, improving cachexia-related symptoms and quality of life, they are unlikely to address all symptoms impacting nutritional intake. Recently, a consensus definition was proposed for Nutrition Impact Symptoms (NIS) [40], based on a survey of international health care providers: *symptoms that compromise patients’ desire or ability to eat, interfering with their nutritional needs and increasing the risk for malnutrition, loss of lean body mass, and impaired QOL. NISs such as nausea, vomiting, early satiety, constipation, depression, anxiety, severe pain, mucositis, and dysgeusia can contribute to decreased caloric intake*. There was a tentative agreement on 24 symptoms that included, but were not limited to, nausea, vomiting, early satiety, constipation, depression, anxiety, severe pain, mucositis, and dysgeusia. Some symptoms were considered less likely to be NIS (short breath, cough, fever, sleep disruption, anxiety, and delirium).

Retrospective studies supporting an association between NIS burden and cancer cachexia-related outcomes are accumulating, underscoring the importance of addressing NIS. The aggregate number of NIS are shown to correlate with survival [41], quality of life [42], degree of weight loss [43], inability to return to work [44], and lower performance status. The severity of the individual symptoms also appears to affect outcomes. A study evaluating 19 symptoms in 302 oncology patients found a higher number of NIS with a score ≥4/10 correlated with lower nutritional intake [45]. NIS are more common in older patients [46] (>50 years old) and are often persistent, so that 46% of patients experience NIS 12 months after chemotherapy [47]. The specific pharmacologic agents may vary among countries depending on availability of medications. The specific drugs proposed for NIS include medications that may benefit more than one symptom or a symptom cluster [48], e.g., olanzapine. Olanzapine has evidence to support its effects in preventing chemotherapy-induced nausea and vomiting (CINV) [49], as well as non-CINV [50] in patients with cancer, major depression, and is recommended by the American Society of Clinical Oncology (ASCO)’s cachexia guidelines for poor appetite [51]. The current ASCO recommendation is supported by two RCTs showing that olanzapine improves appetite and weight significantly compared to placebo in patients with advanced solid tumors [52,53].

More broadly, addressing NIS with an interdisciplinary team may be the most effective approach, given the benefit demonstrated by the open-label phase III trial in patients with metastatic esophageal cancer. Early interdisciplinary care by a team of GI medical oncologists, oncology nurse specialists, dietitians, and psychologists improved median overall survival (14.8 vs. 11.9 months) [6]. Improvements were noted in cachexia-related outcome measures such as the PGSGA and weight compared to the group receiving only standard care.

## 6. Exercise

Exercise may benefit patients with CC by modulating muscle metabolism, insulin sensitivity, hypogonadism, and systemic inflammation. Exercise interventions improved lean mass, QoL, and fatigue. A systematic review found [54] exercise to be safe, to improve quality of life, and to be beneficial for muscular and aerobic fitness both during and after treatments for cancer. Despite limited studies in patients with more advanced, incurable cancer, a systematic review identified improved physical endurance and depression scores among the eight studies of 685 patients [54]. A feasibility trial that randomized 45 patients to either a personalized exercise and nutrition-based program (experimental arm) or standard care (control arm) for 8 weeks demonstrated 80% adherence and decreased health care utilization. This approach of combining nutrition and exercise as an intervention is intuitive; however, there are few studies published. A systematic review and meta-analysis to determine the effect of exercise and nutrition interventions in patients receiving HSCT found 11 studies using exercise interventions and two nutrition interventions; no study used a combined intervention. Meta-analysis of the trials using exercise intervention showed statistically significant effects on 6 min walk, lower extremity strength and global quality of life [55].

## 7. Nutrition

Guidelines from professional organizations note that dietary counseling [51] (ASCO), nutrition intake, calories and protein (ESPEN) [56] are important for achieving optimal clinical results in oncology patients. ESPEN guidelines recommend a protein intake of 1.0 to 1.5 g/kg/day for oncology patients to maintain or restore lean body mass, a challenging target [57] particularly in older patients [58]. However, while some studies suggest that nutritional interventions may improve weight or dietary habits, a systematic review concluded that the evidence remains inconclusive [59]. Specific nutritional supplements have also not consistently improved clinical outcomes in multi-center trials. A systematic review of nutritional interventions identified some supplements, e.g., eicosapentaenoic acid (EPA) and β-hydroxy-beta-methyl butyrate (β-HMB), producing weight gain but overall, the 26 studies reviewed were remarkable for the many trials showing no benefit [60]. Although the evidence is still limited for specific nutritional interventions in cachexia, there are systematic reviews demonstrating nutritional interventions decrease post-acute health care utilization and re-admissions [61].

Despite the critical role of nutrition, parenteral administration has not improved outcomes in a variety of patients with cancer, including those receiving chemotherapy. However, large observational studies have reported that home parenteral or enteral artificial nutrition may prolong survival and improve performance status [62]. An updated systematic review of complications related to enteral nutrition (EN) and parenteral nutrition (PN) found complication rates were equivalent, but EN showed ‘marginal’ superiority for infection among adults [63]. In a prospective randomized trial of patients with cachexia and no intestinal impairment, PN did not improve quality of life and was associated with more serious adverse events than oral nutrition among patients with advanced cancer [64]. Another prospective study reported that survival has not improved over the past two decades in patients with gastrointestinal or gynecologic cancer and co-existing intestinal failure [65].

More randomized clinical trials are needed to identify optimal interventions for oncology patients with cachexia. However, there are challenges in trial design that may be unique including baseline nutritional status of study participants, defining appropriate control groups, and the effective blinding of participants and investigators [66].

## 8. Future Perspectives

The design of clinical trials for anti-cachectic agents should be based on a consistent methodology and outcome measures [4]. However, many of these important issues remain unresolved, including whether specific nutritional support, exercise interventions and optimal symptom management should be mandated for control groups in cachexia trials. Emerging issues include the increased use of Glp-1 agonists and their potential benefit in decreasing obesity and mediating an anti-neoplastic effect. Balancing these positive clinical outcomes with the possibility of exacerbating muscle loss and cachexia are concerns. Given the limited evidence so far, additional research in this area will be needed. Although the highest prevalence of GLP-1 agonist prescriptions is observed among patients with obesity-related cancers (thyroid, breast, and endometrial), the overall use continues to increase among all cancers. Using an electronic medical record database researchers found 4.1% of patients with cancer received at least one prescription for semaglutide or tirzepatide by mid-2025 [67]. Another dilemma, concerning GDF-15 monoclonal antibody agents, may be more welcome. This relates to the anticipated beneficial clinical outcomes of weight, muscle and physical activity and whether they should be attributed to the agent’s anti-cachectic or anti-neoplastic effects.

## 9. Conclusions

Based on some early trials, a personalized multimodal approach may be possible, which is tailored to an individual patient’s needs. A range of clinical targets might include body composition, physical function, serum biomarkers, appetite and other symptoms. A recent Cochrane review of the multimodal interventions for cachexia concluded that methodologically rigorous, well-powered RCTs with adequate interaction times are needed to assess the effectiveness of multimodal interventions in managing cachexia across chronic illnesses [68]. This directive for a rigorous methodology and adequately powered clinical trials may soon be within reach. Once the benefit of individual agents is established in multi-center phase III studies in 2026, multimodal studies using a combination of pharmacological and non-pharmacologic intervention would seem to be the appropriate next step in the evolution of cancer cachexia management.

## Figures and Tables

**Table 1:** Chronological overview of clinical trials (completed or active) targeting cancer cachexia.

Trial ID	Title of Study	Phase	Study Population and Setting	Intervention/Treatment	Status	Outcome Measures and Results
NCT01238107	A Clinical Study With MT-102 in Subjects with Cancer Cachexia (2011)	II	Evaluated MT-102 (Espindolol) in patients with stage III/IV colorectal cancer or non-small cell lung cancer-related cachexia. Participants received MT-102 10 mg twice daily or placebo for 16 weeks. The primary endpoint was the rate of weight change over 16 weeks.	**Espindolol** (Atypical β blocker with a unique, multi-action profile: reduces muscle breakdown, promotes muscle preservation/building, and may improve appetite and fatigue via central nervous system effects.	Completed	Espindolol reversed weight loss, increased lean body mass, and better-preserved muscle strength compared with placebo over 16 weeks.
NCT02767557	Study of Nab-Paclitaxel and Gemcitabine with or Without Tocilizumab in Pancreatic Cancer Patients (PACTO) (2016)	II	Evaluated a chemotherapy combination with or without an immunotherapy drug for patients with locally advanced or metastatic pancreatic cancer (unresectable pancreatic carcinoma). The study was randomized and aimed to assess whether adding an anti-inflammatory/immune-modulating agent could improve outcomes over standard chemotherapy alone.	**Tocilizumab** Anti-IL-6-receptor antibody in combination with chemotherapy (gemcitabine and nab-paclitaxel)	Completed	Primary endpoint: adding tocilizumab failed to significantly improve 6-month overall survival compared with chemotherapy alone. Secondary analyses suggested potential benefits, including improved longer-term survival at 18 months and reduced muscle loss.
NCT04301765	Improving Cancer-related Fatigue, Sexual Dysfunction and Quality of Life in Older Men with Cancer and Androgen Deficiency (TEMEC) (2020)	III	Study of physiological testosterone replacement for cancer-related fatigue, sexual dysfunction, and quality of life in men over 55 with solid or hematologic cancers and low testosterone.	**Testosterone gel** 1.62% daily self-application	Recruiting	Primary: patient reported outcome FACIT-fatigue. Secondary: sexual function; mood and well-being; body composition by DEXA; physical activity; and sleep quality. Exploratory: 6 MW and muscle strength.
NCT04725474	First-in-Human Study of the GDF-15 Neutralizing Antibody Visugromab (CTL-002) in Patients with Advanced Cancer (GDFATHER) (2020)	I/II	Intravenous (IV) administration of CTL-002 given as monotherapy and/or in combination with an anti-PD-1 checkpoint inhibitor in patients with advanced-stage, relapsed/refractory solid tumors.	**Visugromab** GDF-15 mAb Anti-PD-1 checkpoint inhibitor	Active	Preliminary evidence of clinical benefit with improved tumor responses and indications of weight gain or cachexia mitigation in subgroups.
NCT05243251	Olanzapine for Anorexia Cachexia (2021)	III	Incurable solid tumor, loss of appetite score ≥4 on a 0 to 10 numeric rating scale. Cachexia (loss of >5% body weight over last 6 months or any weight loss >2% with a body mass index <20 kg/m^2^	**Olanzapine** 5 mg at night	Completed	ESAS-Anorexia score after 1 week significantly better in olanzapine group. After 4 weeks, more in olanzapine group had >5% weight gain (14% vs. 0%; *p* = 0.008). Anxiety, insomnia, and nausea better. Anemia, leukopenia and decreased Grip strength more common in olanzapine.
NCT05546476	Study of the Efficacy and Safety of Ponsegromab in Patients with Cancer, Cachexia and Elevated GDF-15 (PROACC-1) (2022)	II	Double-blind study to evaluate the efficacy, safety and tolerability of ponsegromab compared to placebo in patients with active diagnosis of non-small cell lung, pancreatic, colorectal cancer with cachexia defined by Fearon criteria and elevated serum GDF-15 concentrations.	**Ponsegromab** (GDF-15 mAb)	Completed	Significant increase in body weight vs placebo at 12 weeks across dose levels. Improvements in appetite, cachexia symptom scores, and physical activity observed especially at highest dose. Open-label extension data suggest sustained weight gains up to ~5.2 kg by 64 weeks.
NCT06989437	Phase 2b/3, Randomized, Double-Blind Study to Investigate Efficacy, Safety, and Tolerability of Ponsegromab Compared with Placebo with Background First-Line Chemotherapy in Adults with Cachexia and Metastatic Pancreatic Adenocarcinoma (2025)	IIb/III	Investigate the efficacy, safety and tolerability of systemic chemotherapy plus ponsegromab versus systemic chemotherapy plus placebo for the first-line treatment in adult participants with cachexia and mPDAC. The first-line chemotherapies will either be nab-paclitaxel plus gemcitabine or FOLFIRINOX (or mFOLFIRINOX). The double-blind period is followed by an optional open-label extension period.	Combination Systemic Chemo + **Ponsegromab** (GDF-15 mAb)	Recruiting	Primary: Body weight and FAACT 5 at 12 weeks. Secondary: physical activity, overall survival, PFS, body composition, adverse events, PROMIS physical function and fatigue; chemo dosing changes, and RECIST.
NCT07112196	VINCIT. Visugromab in Cachexia International Trial (2026)	II/III	Multi-center randomized controlled trial of visugromab in patients with weight loss and advanced cancer visugromab at low, medium, high dose, and placebo. Patients with advanced NSCLC or colorectal cancer receiving chemo or immunotherapy. Upper GI and pancreatic cancer only in Phase II.	**Visugromab** GDF-15 mAb	Recruiting	Primary: body weight. Secondary: FAACT 5 skeletal muscle mass/index by CT/MRI; function by chair stand test; physical activity monitoring; overall survival; tumor response; patient global impression of severity and change; and EORTC-QLQ-C15-PAL.

## Data Availability

The original data presented in the study are openly available in PubMed at http://pubmed.gov and ClinicalTrials.gov at https://clinicaltrials.gov/.
